# Examining the role of firearm involvement in repeat intimate partner violence assaults

**DOI:** 10.1186/s40621-024-00492-7

**Published:** 2024-03-04

**Authors:** Zainab Hans, Chiara E. Cooper, April M. Zeoli

**Affiliations:** 1 Institute of Firearm Injury Prevention, University of Michigan, Ann Arbor, MI USA; 2 School of Public Health, University of Michigan, Ann Arbor, MI USA

**Keywords:** Intimate partner violence, Firearms, Recidivism, Survival analysis

## Abstract

**Background:**

Intimate partner violence (IPV) remains a pervasive and complex issue with significant social and public health implications. The nexus of firearms and intimate partner violence (IPV) is an especially dangerous one. However, little is known about how firearm involvement can influence the risk of repeat IPV assaults.

**Methods:**

We use data from 346 male perpetrated IPV incidents reported to the Detroit Police Department between December 2016 and April 2017 to examine the role of firearm involvement in IPV recidivism during a 5 and half year follow up period. Employing a conditional gap-time frailty model that accommodates heterogeneity among individuals through a frailty term, we analyze time to multiple IPV assaults that occur over the follow up period. We identify various pathways through which firearms impact the likelihood of subsequent IPV incidents, including intimidation, threats, and use of firearms, while controlling for observable perpetrator characteristics to understand the explicit roles of firearms.

**Results:**

Firearm involvement at the index assault was not associated with IPV recidivism. However, involvement of firearms in past IPV assaults significantly increased the risk of subsequent physical IPV. The discrepancy is likely arising from a high degree of censoring among individuals who were armed with a firearm during the index assault.

**Conclusion:**

Our research reveals a nuanced relationship between firearm involvement and IPV recidivism, shedding light on the multifaceted dynamics at play. By elucidating the intricate dynamics at the intersection of firearms and intimate partner violence, our study underscores the need for targeted policy interventions and preventative measures aimed at reducing IPV recidivism.

## Background

Intimate partner violence (IPV) is a persistent public health problem (Basile et al. 2022). Data suggests that almost 1 in 2 (47.3% or 59 million) women in the United States report experiencing sexual or physical violence, and/or stalking by an intimate partner at some point in their lifetime (Basile et al. 2022). Estimates of non-fatal firearm use against women show that nearly 4.5 million women in the United States have been subjected to threats by their partners, and approximately 1 million have been targeted in assaults where the firearm was discharged (Sorenson and Schut 2018). The presence of a firearm in abusive relationships is associated with subsequent lethal outcomes (Campbell et al. 2003; Spencer and Stith 2020), making firearm violence that occurs in the context of IPV a particularly serious issue.

Recent research has provided insight into understanding the nuanced ways violent partners use firearms. However, gaps exist regarding the role firearms play in repeat perpetration. Individuals who are violent towards their partners and also possess firearms may have a higher propensity to engage in more severe or protracted abuse (Folkes et al. 2013; Sorenson and Wiebe 2004). Conversely perpetrators’ access to firearms may shift the balance of power in an abusive relationship in a manner that emboldens them to engage in more frequent abuse.

A significant body of literature has been devoted to identifying and understanding risk factors associated with nonfatal and fatal IPV perpetration (Campbell et al. 2003; Reingle et al. 2014; Sijtsema et al. 2020; Spencer and Stith 2020; Wymbs et al. 2017). Among these, firearms have been seen as incontestably dangerous (Campbell et al. 2003; Sheppard et al. 2022). Evidence suggests that access to firearms increases the likelihood of violence turning deadly in intimate relationships (Campbell et al. 2003; Sheppard et al. 2022; Spencer and Stith 2020). The risk posed by firearms in IPV situations disproportionally affect women. Studies examining the nexus of IPV and firearms have found that the majority of non-fatal firearm-involved IPV incidents are perpetrated by male partners (Sorenson 2017) against female victims (Lyons et al. 2022; Tjaden and Thoennes 2000; Addington and Perumean-Chaney 2014). These gender differences are also apparent in the most extreme manifestation of IPV. Research shows that 44% of homicides committed against women are done so by an intimate partner with firearms being the predominant weapon used (Fridel and Fox 2019). Moreover, intimate partner homicides (IPH) involving firearms have been increasing since 2014, while non-firearm IPH does not mirror this trend (Fox 2021).

In non-fatal contexts, firearms may be used to threaten and intimidate a victim. They may be brandished to communicate non-verbal threats and incite terror (Logan et al. 2022; Lynch and Logan 2018; Sorenson 2017; Sorenson and Wiebe 2004; Sorenson and Schut 2018). Further, a firearm may simply be displayed around the home in plain sight for the victim to see. The abusive partner does not have to explicitly threaten firearm use in such instances. The threat of extreme violence is deducible from the firearm’s presence, and can create environments of extreme fear (Tutty 2015; Lynch and Logan 2018; Sorenson and Schut 2018). Use of firearms in these subtle, nuanced ways can serve an instrumental role in strategically coercing the victim (Sorenson and Schut 2018), and maintaining pervasive control over them (Kafka et al. 2021; Logan et al. 2022; Valente and Graber 2021). Take for instance a victim whose partner is controlling in all aspects of her life, from digitally surveilling her to demanding she wear certain clothing. This same partner has access to a gun. Not only does it render the victim in a constant state of terror, but also creates an intensely hostile and oppressive environment. By making the perpetrator’s threats more credible and increasing perceptions of risk, firearms can create entrapment (Downes et al. 2019; Barlow and Walklate 2021) and facilitate coercive control^1^ (Logan et al. 2022; Logan and Landhuis 2022), leading to protracted cycles of violence (Lynch and Logan 2018; Sorenson and Schut 2018; Tam et al. 2016). As such, non-fatal firearm use can inflict significant harm, instill fear, and have repercussions of victims’ well-being (Tutty 2015; Lynch and Logan 2018; Sorenson and Schut 2018).

While exposure to more severe and frequent IPV can increase the likelihood of help-seeking (Logan and Landhuis 2022; Logan et al. 2022), firearm violence entails an interesting dichotomy. Research shows that women who are subjected to non-lethal firearm violence or whose abusers’ have access to firearms are more likely to report IPV incidents to the criminal justice system (Logan and Landhuis 2022; Logan et al. 2022). However, intervention remains complicated as many perpetrators use firearms to convey subtle non-verbal threats by simply displaying the weapon. Because such incidents do not entail visible injuries and explicit threats (Sorenson 2017), there is no evidence to support allegations of abuse and perpetrators are likely to evade arrest and retain their weapons (Small et al. 2019); which can presumably make victims more vulnerable to future assaults.

One study focusing on a large cohort of firearm purchasers in California, shows that those with prior histories of IPV violence are much more likely to engage in subsequent IPV (Tomsich et al. 2022). However, few studies have utilized information on recurrent IPV incidents (Lyons et al. 2019) and there is a dearth of knowledge regarding the specific role of firearms in repeat IPV. Our paper explores how firearm involvement influences patterns of IPV recidivism, focusing on the frequency and timing of repeat IPV assaults using a longitudinal panel of police reported incidents. We hypothesize that firearm involvement increases the risk of recurrent IPV and test this through a survival analysis approach using data from police incident reports to assess perpetration of repeat IPV assaults, henceforth referred to as recidivism, over a 5 year follow up period. Our study contributes to the existing literature by elucidating the impact of firearm involvement on repeat IPV assaults. We employ a robust methodological approach that partially mitigates some threats to validity that plague empirical studies using observational data. Specifically, we accommodate unobserved heterogeneity among individuals through inclusion of a random effects term to control for individual susceptibility to failure (also referred to as frailty in survival analysis literature); and account for event dependence between repeat incidents by conditioning on the number of previous IPV assaults as we evaluate whether involvement of firearms contributes to a reduction in the time intervals between successive reports of IPV offenses.

## Data and methods

### Study setting

The data come from the Detroit Police Department in Michigan. The city of Detroit has undergone intense change over several decades. Exclusionary housing policies and chronic disinvestment has left the city highly fragmented based on class and race (Desan 2014), with the majority of the city of Detroit’s residents reported to be of Black or African American origin (77.9%) (U.S. Census Bureau 2021).^2^ This demographic make-up of the city reflects in the police reports included in our analysis with Black men constituting majority of the perpetrators. Higher than average unemployment rates (Felson et al 2022) resulting from further economic crises coupled with alarming poverty (U.S. Census Bureau 2021) have created a backdrop against which violence has thrived. Detroit has one of the highest rates of gun violence in the nation (Grommon et al. 2017). Homicides in the city increased nearly 26% from 2019 to 2020 (NIBRS 2020).

Individual level characteristics interact with these structural underpinnings to perpetuate  IPV, especially among those from diverse backgrounds (Sokoloff and Dupont 2005). Research suggests that unemployment, housing insecurity, and financial hardships are factors associated with IPV (Kaukinen and Powers 2015; Doyle and Aizer 2018; Bhalotra et al. 2021). Perpetrators can induce and exploit economic dependency to entrap victims in violent relationships (Littwin 2012). Further, IPV incidents, except the most violent ones, are treated as misdemeanor offenses and therefore, do not impose a significant disutility or sanctions on the perpetrator (Sloan et al. 2013; Visher et al. 2008). If through fear of reprisal or manipulating the victim (Bonomi et al. 2011), perpetrators can effectively exercise some control over the probability of conviction, this can presumably induce a behavioral response that increases repeat incidents of IPV.

Scholars have approximated that Detroit sees 1000 IPV-related police reports per month, demonstrating the need to study intimate violence in this city (Weisz and Schell 2020). We conduct a secondary analysis of a dataset containing information on 346 cases of intimate partner violence reported to the Detroit Police Department between December 2016 and April 2017. Perpetrators were followed until September 2022 to capture any subsequent IPV assaults reported to the police. This research was reviewed and approved by the University of Michigan’s Institutional Review Board.

### Variables

Data were originally collected using the Ontario Domestic Assault Risk Assessment (ODARA) (Hilton et al. 2004), with the inclusion criteria set to include incidents where a male perpetrator physically assaulted or threatened a current or former dating, cohabiting, or married female partner. The 13 empirically selected ODARA items collect information on situational and relationship factors, as well as factors pertaining to perpetrator’s previous criminal and IPV history to assess the risk of IPV recidivism. Because ODARA was designed for use by front line responders, the instrument focuses on information that is readily available to law enforcement. Specifically, the instrument includes questions about violation of conditional release orders (i.e. probation and parole orders), history of incarceration, substance use, prior history of IPV, and violence against other non-IPV individuals. Previous research has found these perpetrator characteristics to be associated with family violence (Hilton et al. 2004; Romero-Martínez et al. 2019; Stansfield et al. 2020; Storey et al. 2014; Williams and Stansfield 2017), therefore we include these as covariates in our analyses. In addition to the original ODARA items, we also collected data on additional items such as economic abuse; use of weapons; and non-fatal strangulation using police reports since these are associated with repeat IPV incidents (Littwin 2012; Mcquown et al. 2016). Non-fatal strangulation, specifically, is a risk marker for homicide and severe assault (Campbell et al. 2003; Messing et al. 2018). Since ODARA allows for item missingness in up to 5 of its 13 items, only 346 cases included in the original dataset had complete information for all the covariates and therefore the present analysis was limited to these cases. To measure recidivism, we code all IPV offences committed by the perpetrators of index assaults that were reported to the police subsequent to the index assault until September 2022, yielding a follow-up period of over 5 years. For individuals that had multiple assaults between December 2016 and April 2017, the earliest incident was selected as the index assault and all subsequent assaults were treated as recidivistic incidents.

Information regarding the index assault was collected directly from police incident reports, which are generated after police officers respond to a call for service and determine a crime has taken place. Reports are usually created by entering data directly into the department’s record management system sections for demographic information on the victim(s), perpetrator(s), witness(es), and any additional individuals involved in the incident, along with a narrative section. The RMS module also contains a section to identify the relationship between victim and perpetrator. Relationship categories include (1) married, (2) formerly married, (3) child in common, (4) dating but not cohabiting, (5) dating and cohabiting, (6) formerly dating. Although in reality multiple categories (i.e. married and child in common) can apply to the victim and perpetrator, only one can be selected in RMS, making these categories mutually exclusive. Police officers are required to enter the circumstances of the crime, as well as whether the victim sustained any injuries, the mechanism of injury, and if the perpetrator was armed with a weapon during the assault and include this information in the narrative section. Officers are also trained to document any “excited utterances” verbatim in their narratives as they are valuable to prosecution as non-testimonial statements and can shed light on the underlying dynamics between the victim and the perpetrator that may have precipitated the assault.

The RMS system consolidates all records pertaining to an individual under a single profile, however, erroneously entered information can lead to creation of multiple profiles. Therefore, wild card searches that allow for partial information to be searched were conducted on each perpetrator. This search can be conducted using partial first and last names and narrowed down by year of birth. For instance, the results for a search term “Tim” yield Tim; Timothy; and Fatima. The search process was repeated for any aliases uncovered and meticulous cross-referencing across all identifying information mitigated the human errors in reports to a large extent, allowing us to link police and court records for each perpetrator. A small proportion of individuals were not observed during follow-up (in other words censored) because of death or incarceration.

Informed by prior literature on the nexus of firearms and IPV (Tutty 2015; Lynch and Logan 2018; Sorenson and Schut 2018), we do not limit our definition of non-fatal firearm use to incidents where the weapon was used. Instead, we operationalized a dichotomous variable as 1 if the narrative indicated the perpetrator was in possession of a firearm and made explicit threats to the victim. To further explore the heterogeneity in firearm involvement, we also code another variable as 1 for incidents where the perpetrator used the firearm to either fire shots or pistol whip the victim. Finally, we also consider cases where the perpetrator was armed and simply displayed the weapon to accurately capture the nuance in non-fatal firearm abuse. For instance, if during an argument, the perpetrator retrieved a firearm from where it was stored and armed himself or placed it in view of the victim, a dichotomous variable was coded 1 because the threat is implied. A separate dichotomous variable was coded as 1 if records of prior IPV assaults indicated that a firearm had been involved and 0 otherwise. Because the involvement of firearms in subsequent incidents can impact the risk of further assaults, we control for the presence of firearms in recidivistic incidents through a binary variable coded as 1 if a firearm was present and 0 otherwise.

A longitudinal history of all cases involving the perpetrator prior to the index assault going back to January 2005 was evaluated to code risk factors for repeat assaults. We searched case narratives to establish any prior assaults committed by the perpetrator against any intimate partner that involved non-fatal strangulation, coding a binary variable as 1 if the perpetrator had strangled the victim and 0 otherwise. Police reports were also used to determine if the perpetrator had assaulted individuals other than an intimate partner to capture an individual’s general proclivity for violence. Leveraging information on whether the perpetrator was intoxicated during the index assault, as well as information on prior arrests in conjunction with drunk driving and possession of dangerous drugs extracted from police reports and court records, a dichotomous variable use was coded as 1 if more than one indicator of substance use applied to the perpetrator (Hilton et al. 2004).^3^ We also coded a dichotomous variable to indicate if the perpetrator had been arrested for index assault. Arrests were either made by responding officers at the time of the incidents or later through warrants. However, arrests refer to booking only as many perpetrators arrested through warrants were not subsequently convicted.

Previous literature suggests that non-fatal firearm violence and coercive control often tends to co-occur and being subjected to multiple forms of abuse can make victims vulnerable to more frequent violence by creating entrapment (Barlow and Walklate 2021; Downes et al. 2019; Logan et al. 2022; Logan and Landhuis 2022). Therefore, we determine if the perpetrator employed other forms of coercive control such as economic abuse. Economic abuse was determined through perpetrator involvement in police reported incidents of malicious destruction of property, arson, and stealing/robbing a partner of economic resources (e.g., money or a vehicle), prior to or in conjunction with the index assault. A dichotomous variable coded 1 if police reports indicate the presence of any of the aforementioned factors and 0 otherwise.

Information regarding incarceration was collected from the Michigan Department of Corrections’ (MDOC) Offender Tracking Information System (OTIS) and 3rd Circuit Court’s Odyssey platform. A dichotomous variable was coded 1 if the records indicated that the perpetrator had been sentenced to at least a 30-day custodial sentence prior to the index assault. Records also indicate if a perpetrator had ever violated conditional release orders (e.g. terms of parole or probation). This information was used to code a separate dichotomous variable as 1 if prior to the index assault, the perpetrator had violated such conditions. If available records did not indicate that a risk factor was present, the variable was coded as 0. While this is a reliable strategy for coding risk factors such as violation of probation and parole order and imprisonment, it may underestimate substance use and economic abuse.

## Modeling strategy

We specify a conditional frailty model to evaluate gap times between repeat incidents of IPV to assess whether non-fatal firearm use during the index assault increases the hazard for recurrent IPV. We adopt this approach because timing of recidivistic incidents within individuals is likely correlated such that the probability of a subsequent assault is influenced by the prior assault and overall risk changes as the number of assaults increase (Dowling et al. 2021). Therefore, we expect our data to be simultaneously characterized by heterogeneity and event dependence, which raises concerns about violation of the independent events assumption and therefore biased results due to underestimated standard errors.

The conditional frailty model combines a frailty term with a stratification approach (Balan and Putter 2020; Box-Steffensmeier and Boef 2006; Box-Steffensmeier et al. 2007) to estimate the effect of firearm involvement on repeat IPV assaults. The model is semi-parametric and makes no assumptions about the baseline hazard. The frailty term represents the between individual heterogeneity and acts multiplicatively on the hazard such that individuals with higher frailty than have higher hazards and shorter survival times. Similar to the Cox Proportional Hazard model, proportionality is assumed conditional on frailty (Balan and Putter 2019), covariates, and strata. Mathematically the hazard in this model can be expressed as

1 $$\lambda_{i} \left( {t{|}Z} \right) = Y_{i} \left( t \right)Z_{i} exp\left( {\beta^{T} x_{i} \left( t \right)} \right)\lambda_{0s} \left( {t - t_{s - 1} } \right)$$

where the subscript *i* represents each individual and $x_{i}$ represent a vector of covariates pertaining to individual* i*, which can be time varying as indicated by *t*. $Y_{i}$ represents if the individual is at-risk of assaulting an intimate partner at time *t*, assuming a value of 1 if individual *i* is in the risk set at *t* and 0 otherwise, and $Z_{i}$ is the unobserved random effect that captures individual heterogeneity. Where $\lambda_{0s}$ is the baseline hazard or intensity that varies by the strata *s* (or equivalently event number), to which individual *i* belongs. In the gap time specification, time is measured until a subsequent IPV assault for individuals with multiple incidents and resets for each stratum. Events are sequential so the risk set for *s* is restricted to individuals who perpetrated the (*s* − 1)th assault and $\lambda_{0s}$ represents the risk for the subsequent sth assault since the previous (s − 1)th incident. Although the maximum number of recidivistic events in our data go up to 14, the number of individuals in higher strata is very small and four or more incidents are collapsed into a single stratum to ensure reliable estimates.

$Z_{i}$ is assumed to be independent and identically distributed such that the timing of the events are independent given $Z_{i}$ (Balan and Putter 2019). We select the gamma distribution for expositional reasons and computational ease as our preferred distribution. This choice is in line with previous empirical work (Abbring and Van Den Berg 2007; Box-Steffensmeier et al. 2007). The analysis is conducted in R using the *survival* and *FrailtyEM* packages (Balan and Putter 2019). The time elapsed between the index and recidivistic incident was measured in number of days. Perpetrators were right censored after September 2022.

## Results

Police reports included in our analysis show that 94.1% of the perpetrators are African American, 2.5% are white, and 3.4% are other minority races like Hispanic and Arab. The average age of the perpetrators is 33 years old. Firearms were involved in nearly 10% of the index assaults. Among individuals that were armed with a firearm during the index assault, 75.7% percent made explicit threats to the victim, 48.5% pointed the firearm at the victim or used it during the assault, and 18.2% used the firearm to either pistol whip the victim or fire shots. Police narratives highlight harrowing accounts of non-fatal firearm use that did not entail any physical injury. For instance, one victim reported that the perpetrator had stuck a handgun in her mouth and pulled the trigger. While the chamber was empty and the victim did not sustain any physical injury, the narrative described her as being in a “state of extreme terror”. Other reports indicated that perpetrators had a firearm in their possession during the assault and displayed it during an argument which can be seen as an implied threat.

Nearly 32% of the perpetrators in our cohort committed more than one physical assault against a partner over the follow-up period. Of these, almost 68% recidivated against the index victim. Overall, 132 individuals had more than one subsequent police report for any kind of IPV offense, leading to a total of 1006 incidents nested within 346 individuals with a mean of 1.92 offenses. Perpetrators who were armed with a firearm at the index assault had an average of 2.36 IPV reports compared to 1.87 offenses for those who were not armed. Focusing on just reports of physical assaults, the overall average is 1.56. Perpetrators of firearm involved assaults on average had 1.8 subsequent police reports, while perpetrators of non-firearm assaults had 1.54. Before proceeding to the main analysis, we investigate group differences between individuals who were armed during the index assault and made explicit threats to the victim and those who did not. Table 1 provides descriptive statistics.

**Table 1:** Descriptive statistics

Cohort Characteristics - Perpetrators who employed firearm threats during index assault							
	Total		Firearm threats = yes		Firearm threats = no		χ^2^*, p* value
	N = 346		N = 25		N = 321		
	N	%	N	%	N	%	
*Perpetrator race*							
Black	325	93.93	23	92.00	302	94.08	χ^2^ = 0.23, *p* value = 0.89
White	9	2.60	1	4.00	8	2.49	
Other	12	3.46	1	4.00	11	3.43	
*Perpetrator age*							
18–24	69	19.94	3	12.00	66	20.56	χ^2^ = 6.81, *p* value = 0.08
25–34	141	40.75	7	28.00	134	41.74	
35–44	81	23.41	11	44.00	70	21.81	
45 and over	55	15.89	4	16.00	51	15.89	
*Relationship status*							
Married	80	23.12	6	24.00	74	23.05	χ^2^ = 1.48, *p* value = 0.69
Child-in-common	196	56.64	12	48.00	184	57.32	
Dating	55	15.89	5	20.00	50	15.58	
Separated	15	4.33	2	8.00	13	4.05	
Strangulation	142	41.04	8	32.00	134	41.74	χ^2^ = 0.91, *p* value = 0.34
Substance use	110	31.79	7	28.00	103	32.09	χ^2^ = 0.18, *p* value = 0.67
Custodial sentence	161	46.53	12	48.00	149	46.42	χ^2^ = 0.02, *p* value = 0.88
Economic abuse	169	48.84	9	36.00	160	49.84	χ^2^ = 1.78 *p* value = 0.18
Prior non-IPV assaults	136	39.30	9	36.00	127	39.56	χ^2^ = 0.12, *p* value = 0.72
Prior IPV assaults	240	69.36	18	72.00	222	69.16	χ^2^ = 0.09, *p* value = 0.77
Firearm use in prior IPV assaults	39	11.27	3	12.00	36	11.21	χ^2^ = 0.01, *p* value = 0.90
Firearm use in future IPV assault	18	5.20	3	12.00	15	4.67	χ^2^ = 2.52, *p* value = 0.11
Arrest at index assault	88	25.43	10	40.00	78	24.30	χ^2^ = 3.01, *p* value = 0.08
Violation of conditional release order	179	51.73	12	48.00	167	52.02	χ^2^ = 0.15, *p* value = 0.70

Firearm threats is coded as 1 if the perpetrator was armed and made explicit threats towards the victim. Individual relationship classification are combined into four broad categories

While perpetrators who threatened their partners were more likely to arrested, the difference is not statistically significant at the 5% level (χ^2^ = 3.01, *p* value = 0.08). Both groups are similar in prior IPV history (χ^2^ = 0.09, *p* value = 0.77) as well as violence against non-IPV victims (χ^2^ = 0.12, *p* value = 0.72). Overall, the characteristics of those with firearm threats at index assault are similar to other perpetrators. It is unusual for observational data to yield such similar groups, however the current sample was limited to cases where information on the ODARA items included in the analysis was available. This necessarily restricted the sample to individuals with multiple police reports (IPV or otherwise) because more information was available in these cases to code the ODARA. Individuals who repeatedly come into contact with the police may have shared characteristics, explaining the similarities we observe among groups. Having a cohort that is similar in observable risk factors allows us to delineate the effect of firearm involvement as an independent risk factor without relying on matching techniques to ensure comparability between control and treatment groups.

We verify the proportionality assumptions using the zph function in R. Panel A of Table 2 provides the results of our gap time conditional frailty model for all IPV offenses reported to the police, including subsequent police reports of non-physical abuse such as intimidation, stalking, theft, and damage to property. The main variable of interest is firearm threats. A likelihood ratio test ensures that the model containing frailty terms is preferable (χ^2^ = 496.9, *p* value = 0.00).

**Table 2:** Time to recurrent IPV events

	Conditional gap-time frailty model		Conditional gap-time model	
	Hazard ratio	Confidence interval	Hazard ratio	Confidence interval
*Panel A: all police reported offenses*				
Firearm threats at index	1.22	0.76–1.98	1.23	0.92–1.65
Firearm involvement in past IPV	1.40	0.96–2.02	1.25	1.02–1.54
Firearm involvement in recidivism	0.99	0.70–1.39	1.06	0.78–1.44
N	1004		1004	
Number of failures	660		660	
Log likelihood	− 3117.51		− 3317.98	
Wald χ^2^	96.47***		90.48***	
I-likelihood	− 3302.63		–	
Θ	0.68***		–	
Likelihood ratio for Θ	400.93***		–	
*Panel B: police-reported physical assaults*				
Firearm threats at index	1.07	0.55–2.08	1.23	0.89–1.70
Firearm involvement in past IPV	1.90	1.13–3.21	1.45	1.15–1.82
Firearm involvement in recidivism	1.03	0.69–1.53	1.07	0.76–1.51
N	886		886	
Number of failures	540		540	
Log likelihood	− 2351.12		− 2621.08	
Wald χ^2^	194.90***		154.67***	
I-likelihood	− 2590.41		–	
Θ	1.36***		–	
Likelihood ratio for Θ	539.93***		–	

Models control for perpetrator characteristics including age, substance use, prior incarceration, history of non-IPV assaults, history of strangulation, violation of conditional release order, relationship type, economic abuse, prior IPV history, and arrest at index assault

*** denote p<0.001

The variance of the frailty term is statistically significant (*p* value = 0.00), indicating variance that cannot be explained by the comprehensive set of covariates included in the model. The coefficients are estimated as log intensity ratios and a value greater than zero represents an increase in relative risk. Exponentiating the beta coefficient of firearm involvement during index assault in conditional gap time frailty model reveals that the risk of recidivism is 1.22 times greater than the baseline risk, but this effect is not statistically significant (95% CI : 0.75–1.97). The effects of firearm involvement in past or subsequent IPV incidents are also not significant. Panel A of Table 2 also includes the results from a conditional gap-time model for comparison. When unobserved heterogeneity is not accounted for, the effect of firearm involvement in recidivistic incidents is opposite in direction showing that failure to account for differences within individuals can result in biased estimates. In contrast to the frailty model the effect of firearm involvement in prior IPV assaults is significant in the conditional gap-time model (HR: 1.28, 95% CI 1.02–1.54), however the magnitude is smaller and the 95% confidence interval is close to 1. The effect of firearm involvement in subsequent IPV offenses is not significant in either model.

Because several categories of offenses included in panel A may be prone to underreporting (e.g. theft). We consider physical violence separately. Panel B presents the results of physical IPV assaults that include aggravated and simple assaults; kidnappings, sexual assaults; as well as any assault where victims were threatened with a firearm. The frailty term is larger than in panel A indicating that there is greater unexplained variance that is unrelated to the included covariates but influences physical violence. Firearm threats during index assault raise the risk of recidivism relative to baseline but the effect is insignificant (HR: 1.065, 95% CI 0.55–2.08). It is important to note that of the perpetrators with firearm threats at index, 28% (n = 7) were censored because of death or incarceration, and 24% (n = 6) were right censored i.e., had no recidivistic incidents before the study period ended. Among those who were not censored, warrants were issued for 33% of the perpetrators which may have deterred subsequent IPV.

Involvement of firearms in prior IPV assaults is significant and elevates the risk of recidivism (HR: 1.90 95% CI 1.12–3.22). This effect is significant at a 5% level after applying a Bonferroni correction for multiple testing. There are differences in magnitude that are worth noting. When unobserved heterogeneity is accounted for, the risk of subsequent assaults is almost twice whereas it only increases by 1.45 times when we do not account for differences among individuals. Among those with prior history, nearly 18% were also armed at the index assault. Additionally, 13% of these perpetrators also had a firearm during recidivistic incidents. In contrast to perpetrators who threatened their partners at index assault, the proportion of perpetrators with a history of IPV assaults involving a firearm (n = 39) that was either right censored or censored because of death or incarceration was much smaller (20%).

Table 3 in “Appendix” shows results for physical assault models where firearm involvement during index assault was defined as perpetrator being armed and displaying the weapon. These models yield consistent results for prior firearm involvement. Table 4 in “Appendix” considers perpetrators who either fired shots or pistol whipped the victim. Due to the censoring of perpetrators of firearm involved index IPV and the difference between prior and index firearm involvement we conduct a post-hoc power test to see how large a sample size would be sufficient to have a 75% chance of correctly rejecting the null hypothesis at a significance level of 0.05. Given the effect size, our test shows that we would need over 300 individuals per group; indicating that our test is underpowered. To explore the effect of firearm involvement on overall number of subsequent IPV assaults and not just the timing of the recidivistic incidents, we test a negative binomial specification to determine whether non-fatal firearm involvement is associated with higher number of subsequent IPV assaults overall. In this specification, we operationalize firearm involvement as the perpetrator being armed with a gun during *any* IPV incident by grouping together index and past firearm involvement together in a single dichotomous variable. Table 5 in “Appendix” shows that firearm involvement is positively and significantly associated with the overall number of recidivistic IPV assaults.

## Discussion

Given the cyclical nature of IPV, understanding the patterns of repeat victimizations and the role firearms play in perpetuating them can further prevention and risk mitigation. Our analysis estimates the relative risk of recidivism among perpetrators by conditioning on prior IPV incidents and measuring time to each subsequent event by resetting the clock after each event while allowing for unobservable heterogeneity through a frailty term. Distinguishing the effects of heterogeneity and event dependence is substantively important as policy implications may differ. If *only* event dependence drives the effect, then appropriate mitigation efforts that endow victims with needed resources and safety nets may be sufficient to break the cycle of violence in some cases. If on the other hand, certain individuals pose an inherently high risk to victims, then additional strategies such as incapacitation through incarceration and/or removal of firearms may be necessary to ensure victim safety.

Previous research shows that firearms can be used in a variety of ways to strategically to create oppressive environments and gain dominance over those victimized. Therefore, the involvement of firearms in the current study was not limited to causing physical injury to the victim. Rather, our data reflects instances where firearms were used to intimidate or harass a victim, or even displayed in a manner that was perceived as threatening by the victim. We believe that this operationalization captures the nuance of firearm violence that takes place within the context of IPV. The results of our empirical analysis show that prior history of firearm involvement contribute to shorter time intervals between repeat IPV assaults. Specifically, the risk of physical violence increases rather than all IPV offenses. While firearm involvement of any kind at the index incident is not significantly associated with subsequent IPV violence after controlling for risk factors and individual level differences, our model is underpowered to detect this particular effect. Therefore, this particular finding need to be interpreted cautiously. We do find a significant and positive association between firearm involvement in an IPV incident and the total number of subsequent IPV assaults using a negative binomial specification. Taken together the results underscore the harmful effect of firearm involvement in IPV incidents as it pertains to risk of recidivism

Our findings, combined with the fact that firearms increase the risk of fatal outcomes considerably, suggest that firearm involvement in IPV assaults, no matter how subtle, should prompt a protocol that is appropriate and responsive to the risk firearms introduce in IPV situations. First responders should assess for lethality risk in IPV situations and connect victims to supportive resources (Messing et al. 2015). Additionally, it is essential that risk screenings adequately and accurately reflect that firearm abuse can be harmful in ways that go beyond physical injury (Logan et al. 2022). Victims often describe their lived experiences not through frameworks of discrete violent episodes, but rather in relation to the micro-regulation of their everyday lives whereby control is a central dynamic (Stark and Hester 2019; Overstreet et al. 2021). In such situations, the mere presence of firearm may be enough to elicit compliance without resorting to physical violence. Importantly still, because this kind of violence is subtle, it may not *always* be recognized as harm by law enforcement officials because of lack of injury and/or explicit threats. Taking this further, this kind of violence would prove extremely challenging for which to provide compelling evidence in a court setting. It may not even meet the legal definition of an assault because of lack of demonstrable harm, making it a legislative grey area and leaving those victimized who want criminal prosecution with limited opportunities for pursuing legal recourse.

Thus, it is crucial to recognize that the true complexity of this type of nonfatal firearm violence can easily be missed among official statistics and even traditional victimization surveys that focus on the extremes of firearm violence. Individuals tasked with assessing risk should be informed of the various ways that firearms can be used to strategically coerce victims and maintain pervasive control over them. Criminal justice initiatives targeting perpetrators through focused deterrence programs or mitigating risk through firearm relinquishment might also be beneficial for those survivors who have few alternate resources to ensure their personal safety. Early detection and response may also prevent situations where victims facing a multitude of risk factors are too afraid to seek help. Such prevention strategies will be beneficial for the most vulnerable victims, and can, potentially, offer some remedy to the disenfranchisement minority victims feel from the criminal justice system.

We must acknowledge that our study has several shortcomings. The analysis is based on secondary data and may be prone to some reporting errors. While we address the issue of unobservable heterogeneity through our methodological approach, we are mindful that our results are dependent on IPV incidents being reported to the police. Our thorough examination of records allowed us to ensure that lack of subsequent assaults appearing in police data was not simply an artifact of individuals moving to a different jurisdiction, we are still unable to completely address the issue as some victims simply may not wish to report. Weisz and Schell (2020) show that female IPV victims in Detroit often do not obtain protective orders because they perceive that pursuing legal recourse increases their risk of victimization. Furthermore, victims also harbor concerns about institutional racism and are reluctant to subject their partners to a legal system and instead seek alternate avenues of help. These concerns combined with increased risk posed by firearms may result in lower reporting. It is also possible that some subsequent calls for service may not have resulted in a police report if police officers were not able to determine that an assault occurred. Previous research has found that criminal justice system actions such as arrest are protective against repeat IPV assaults. While we control for arrests in our analysis, it may be the case that additional measures such as issuing warrants and proceeding with criminal prosecution also have a deterrent effect. Unfortunately, we do not have warrant information on all cases included in our analysis, preventing us from assessing the impact of measures beyond arrest on IPV recidivism. The Detroit Police Department has increased focus on efforts to reduce firearm violence. Unfortunately, it is outside the scope of the current analysis to control for these broader efforts targeting firearm violence. Lastly, because our study focuses on a specific geographic area, the results are not generalizable. However, to the extent that our cohort reflects perpetrators of similar socio-demographic characteristics, our findings are relevant for IPV prevention. Our results contribute to the body of empirical work investigating the link between firearms and IPV. The methodological approach highlighted in our study can inform future research employing survival analyses by offering simple but effective strategy to account for unobserved heterogeneity and event dependence since neither stratification nor random effects offer a reliable modelling strategy in and of itself when both processes simultaneously characterize the data.One important issue our study highlights is statistical power. Prior research shows that 4.5 million women in the United States have experienced firearm involved IPV (Sorenson and Schut 2018), yielding estimates of under 3% of women who have suffered from such victimization. After accounting for censoring, the number of perpetrators who were armed with a firearm at index assault yields a comparable percentage in our data. Given the low prevalence, future studies using alternate data sources may also encounter similar issues in detecting significant effects. Such results should not be taken to undermine the very real harm inflicted by firearm involvement as statistical significance is not an ideal measure to gauge substantively important differences in small samples.

## Conclusion

Utilizing information on multiple recidivistic incidents we show that non-fatal firearm use during prior IPV assaults is significantly associated with the time interval between repeat IPV assaults. In contrast, firearm use at index assault does not increase the relative risk of future IPV. It is worth noting that despite the high degree of censoring, the average number of recidivistic assaults is higher among perpetrators who were armed at index assault. Therefore, a different dataset that captures victimizations more accurately or where perpetrators are not censored may yield a significant effect of firearm involvement on recidivism. Future research should continue to explore this relationship by focusing on broader samples to provide generalizable inferences as well as considering how various risk factors interact with each other. Given that observational data does not allow for fully controlling for confounding factors, models incorporating a frailty term and multiple events can be a useful alternative to provide a comprehensive picture of the dynamics that characterize abusive relationships.

## Data Availability

The data used in this study was obtained from the Detroit Police Department under a Data Use Agreement and cannot be shared publicly.

## Appendix

See Tables 3, 4 and 5.

**Table 3:** Time to recurrent IPV events among perpetrators armed with a firearm during index assault

Police reported physical assaults				
	Conditional gap-time frailty model		Conditional gap-time model	
	Hazard ratio	Confidence interval	Hazard ratio	Confidence interval
Firearm involvement at index	0.99	0.55–1.80	1.08	0.81–1.45
Firearm involvement in past IPV	1.90	1.12–3.23	1.43	1.14–1.81
Firearm involvement in recidivism	1.03	0.69–1.53	1.07	0.76–1.51
N	886		886	
Number of failures	540		540	
Log likelihood	− 2350.24		− 2621.65	
Wald χ^2^	195.07***		154.09***	
I-likelihood	− 2590.41		–	
Θ	1.37***		–	
Likelihood ratio for Θ	542.83***		–	

Firearm involvement was defined as perpetrator being armed and displaying a weapon. All models control for perpetrator characteristics including age, substance use, prior incarceration, history of non-IPV assaults, history of strangulation, violation of conditional release order, relationship type, economic abuse, prior IPV history, and arrest at index assault

﻿*** denote p<0.001

**Table 4:** Time to recurrent IPV events among perpetrators who used firearms during index assault

Police reported physical assaults				
	Conditional gap-time frailty model		Conditional gap-time model	
	Hazard ratio	Confidence interval	Hazard ratio	Confidence interval
Firearm use at index	2.68	0.83–8.63	2.87	1.79–4.60
Firearm involvement in past IPV	1.89	1.15–3.13	1.49	1.18–1.86
Firearm involvement in recidivism	1.02	0.69–1.52	1.08	0.77–1.52
N	886		886	
Number of failures	540		540	
Log likelihood	− 2390.75		− 2614.31	
Wald χ^2^	197.01***		167.22***	
I-likelihood	− 2588.70		–	
Θ	1.23***		–	
Likelihood ratio for Θ	447.11***		–	

Firearm use is defined as firing shots or pistol whipping the victim. All models control for perpetrator characteristics including age, substance use, prior incarceration, history of non-IPV assaults, history of strangulation, violation of conditional release order, relationship type, economic abuse, prior IPV history, and arrest at index assault

﻿*** denote p<0.001

**Table 5:** Association between non-fatal firearm use and number of recidivistic IPV events - Negative Binomial Specification

Variable	Incidence rate ratio	Confidence interval
Non-fatal firearm involvement	1.48	1.04–2.11
Log likelihood: − 539.84		
Observations: 346		

Estimates are based on a negative binomial model. The outcome variable is the number of total IPV assaults committed after the index assault. Non-fatal firearm involvement is defined as perpetrator being armed by a firearm. The model control for perpetrator characteristics including age, substance use, prior incarceration, history of non-IPV assaults, history of strangulation, violation of conditional release order, relationship type, economic abuse, prior IPV history, and arrest at index assault

## Abbreviations

IPV: Intimate partner violence
IPH: Intimate partner homicide
HR: Hazard ratio
CI: Confidence interval
NIBRS: National Incident Based Reporting System
MDOC: Michigan Department of Corrections
OTIS: Offender Tracking Information System

## Footnotes

1 Stark (2007) identifies coercion as employing force or threats to induce or deter a specific response, while control refers to “structural forms of deprivation, exploitation, and command that compel obedience indirectly” (p. 229; Stark and Hester 2019).

2 Exclusionary housing policies and chronic disinvestment led to the so-called “white flight”, evidence of which is reflected in the City’s demographic makeup. Redlining of the city’s neighborhoods triggered disparities that have resulted in lasting impacts on poverty, economic opportunity, living environments and the health of residents (McClure et al. 2019; Shkembi et al. 2022).

3 ODARA assesses substance misuse on several items, including whether the perpetrator was under the influence of drugs and/or alcohol during the index incident; if the perpetrator had increased the use of drugs and/or alcohol in the days leading up to the index assault; if the perpetrator exhibited more violent/angry behavior while under the influence; if the perpetrator had previously been charged for a criminal offense while under the influence; and whether the perpetrators had had a drug or alcohol problem at any time since he/she had turned 18.
